# Evaluation of Upper Airway Growth Patterns With Cephalometric Analysis Based on Cervical Vertebral Maturation in the Japanese: A Cross-Sectional Study

**DOI:** 10.7759/cureus.70741

**Published:** 2024-10-02

**Authors:** Takayoshi Ishida, Asuka Manabe, Takashi Ono

**Affiliations:** 1 Orthodontics, Koshigaya Ace Dental Clinic, Saitama, JPN; 2 Oral and Maxillofacial Surgery/Orthodontics, Yokohama City University Medical Center, Yokohama, JPN; 3 Orthodontics, Tokyo Medical and Dental University (TMDU), Tokyo, JPN

**Keywords:** upper airway, lateral cephalometric radiographs, cross-sectional area, cervical spine age, cervical vertebral maturation stage

## Abstract

Objective

This study aims to establish standard values for the upper airway cross-sectional area and evaluate growth patterns using the cervical vertebral maturation stage (CVMS) in a Japanese population.

Methods

A cross-sectional sample of 400 patients, aged 6-20 years, was selected randomly from the Orthodontic Clinic at Tokyo Medical and Dental University (TMDU) dental hospital. Cervical vertebral maturation stages (CVMS I-V) guided the classification of participants into five equal groups. Lateral cephalometric radiographs taken prior to orthodontic treatment were used to measure the upper airway's cross-sectional area. The growth spurt and sex differences in growth patterns were assessed through these measurements.

Results

Standard values for the upper airway dimensions at each CVMS stage were established. Significant growth spurts were noted between CVMS II-III and CVMS III-IV in males and at CVMS II-III in females. The weighted kappa coefficient (κ) demonstrated almost perfect intra- and inter-evaluator agreement, confirming the reliability of CVMS in growth assessment.

Conclusion

CVMS provides a reliable framework for assessing growth patterns of the upper airway, with distinct variations between sexes noted. These findings support the utility of CVMS in clinical growth evaluation and orthodontic treatment planning.

## Introduction

Respiration is a vital physiological function for mammals in which air flows into the lungs through the airway. Human growth and breathing are closely related. Previous reports have shown that obstructive sleep apnea (OSA) in children can cause dental malocclusion, abnormal maxillofacial growth modification, physical developmental disorders, emotional anxiety, and learning disabilities [1-4]. OSA is widely recognized as an adult disease but its prevalence in children is reported to be approximately as high as 3% [5].

Occasionally, enlarged adenoids and tonsils in children obstruct the upper airway (UA) and worsen OSA symptoms [6-8]. Based on the growth and development of lymphoid tissue in the body, Scammon et al. reported that lymphoid tissue overgrows to 200% of its original size and then regresses to 100% by adulthood [9]. Based on this analogy, treatment strategies in various medical fields have been devised based on the assumption that the size of lymphoid tissue will eventually decrease after puberty. Therefore, it is believed that the growth curve of adenoids and tonsils is the same as that of Scammon's lymphoid growth curve, although the size of adenoids and tonsils has not been measured over time. However, our group reported that contrary to conventional theory, pharyngeal and palatine tonsils grow slowly, without excessive increase or decrease during the ages of 6 to 20 years [3].

In general, under the same conditions of lung suction, airflow is reduced if the UA is stenotic and increased if it is wide. In terms of improving the respiration environment during growth, it is reasonable to use orthodontic treatment techniques to promote physiological growth of the maxilla and mandible, which play a role in determining the UA size, or to use surgical procedures to enlarge the UA by removing hypertrophied adenoids and tonsils in combination with jaw surgery.

In a previous study, we reported maxillary and mandibular growth patterns with cephalometric analysis based on cervical vertebral maturation in the Japanese population and demonstrated that the cervical vertebral maturation stage (CVMS) is a useful tool for the growth assessment of patients undergoing orthodontic treatment [10]. However, there are few reports on the UA growth in the Japanese population.

Therefore, the purpose of this study was to (1) calculate standard values of the cross-sectional area of the UA in Japanese, (2) evaluate the UA growth pattern in Japanese, and (3) assess the usefulness of the CVMS for UA growth assessment.

This work was previously posted on the Research Square preprint server on April 20, 2022 (https://doi.org/10.21203/rs.3.rs-1505014/v1).

## Materials and methods

Study design and setting

A cross-sectional study was conducted by random sampling of patients who visited the Orthodontic clinic at Tokyo Medical and Dental University (TMDU) Dental Hospital. The setting provided a controlled clinical environment suitable for high-precision orthodontic measurements and included a well-documented patient demographic. The study was conducted over a six-month period from July 2021 to December 2021, during which data collection was carried out following the approved protocols.

Ethics statement

This cross-sectional study was approved by the Ethics Committee of TMDU Dental Hospital (D2021-026). Before participation in the study, patients and their guardians provided written informed consent in accordance with the research protocol approved by the Institutional Review Board.

Participants

**Figure 1:**
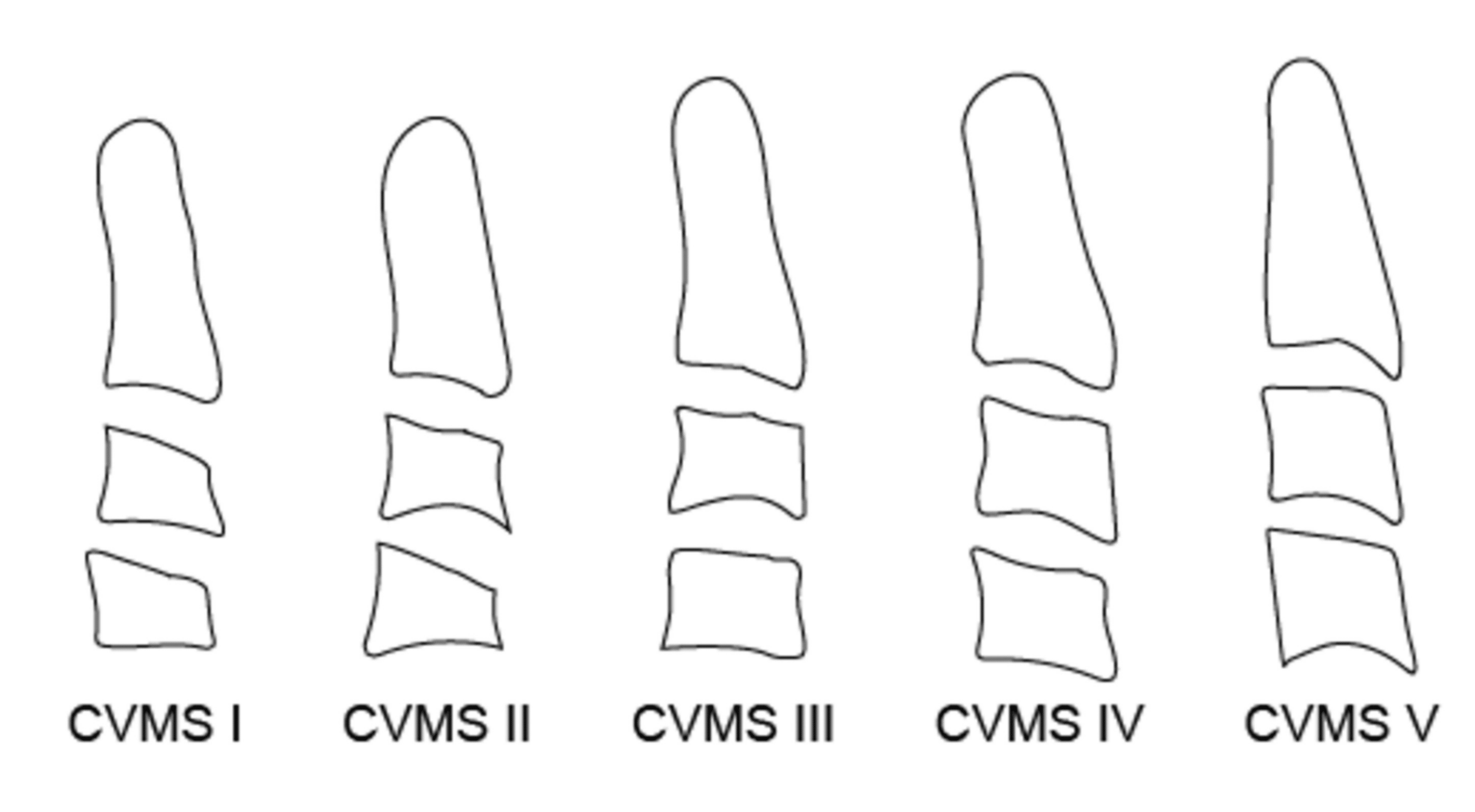
Definitions of cervical vertebral maturation stage (CVMS) CVMS I: the lower borders of three vertebrae are flat. CVMS II: Concavities at the lower borders of both C2 and C3 are present. CVMS III: Concavities at the lower borders of C2, C3, and C4 are present. CVMS IV: At least one of the bodies of C3 and C4 is square in shape. CVMS V: At least one of the bodies of C3 and C4 is rectangular and vertical in shape. This figure is the original work of the authors.

The patients were Japanese and aged between 6 and 20 years. Overall, 4000 patients were assigned random coefficients, and the sample to be measured was randomly selected [11]. In addition, random sampling was repeated until the sample size in each group (n=40) was achieved [11]. Finally, 400 patients participated in this study and were assigned to five male and five female groups (CVMS I-V) based on the CVMS (Figure 1) [12]. Inclusion criteria were patients who had not undergone any surgical treatment that would affect the growth of the jaw or airway and had not had orthodontic treatment yet. Exclusion criteria were syndromes affecting maxillofacial morphology, such as cleft lip and palate, Down syndrome, and Marfan syndrome, and other related disorders that could interfere with the study outcomes (cleft lip and palate, Goldenhar syndrome (including branchial arch anomalies), craniofacial dysostosis, Treacher Collins syndrome, Pierre Robin sequence, Down syndrome, Russell-Silver syndrome, Turner syndrome, Beckwith-Wiedemann syndrome, hemifacial microsomia, congenital myopathy, muscular dystrophy, spinal muscular atrophy, hemifacial hypertrophy, Ellis-van Creveld syndrome, osteogenesis imperfecta, ectodermal dysplasia, neurofibromatosis, basal cell nevus syndrome, Noonan syndrome, Marfan syndrome, Prader-Willi syndrome, facial clefts (including lateral facial cleft, oblique facial cleft, and midline facial cleft), osteopetrosis, albinism, oral-facial-digital syndrome, Moebius syndrome, Kabuki syndrome, Klippel-Trenaunay-Weber syndrome, Williams syndrome, Binder syndrome, Stickler syndrome, microglossia, craniosynostosis (including Crouzon syndrome and Apert syndrome), osteogenesis imperfecta, Freeman-Sheldon syndrome, Rubinstein-Taybi syndrome, chromosomal deletion syndrome, Larsen syndrome, melorheostosis, congenital oligodontia (missing six or more teeth), CHARGE syndrome, Marshall syndrome, growth hormone deficiency short stature, polydactyly syndrome (including XXX syndrome, XXXX syndrome, and XXXXX syndrome), ring chromosome 18 syndrome, lymphangioma, holoprosencephaly, Klinefelter syndrome, pseudohypoaldosteronism, Sotos syndrome, glycosaminoglycan metabolism disorder (mucopolysaccharidosis), fibrous dysplasia, Sturge-Weber syndrome, cherubism, pseudohypoparathyroidism, Ekman-Westborg-Julin syndrome, autosomal duplication syndrome, and other congenital anomalies of the jaw and oral cavity). This approach was designed to limit confounding variables and focus on the natural variation in cervical vertebral maturation and its correlation with upper airway development. The descriptive characteristics of the patients are summarized in Table 1.

**Table 1:** Descriptive statistics of patient variables S.D., standard deviation; M, male; F, female; FMA, mandibular plane to Frankfort-Horizontal plane; SNA, sella-nasion to point A; SNB, sella-nasion to point B; CVMS, cervical vertebral maturation stage

	CVMS I		CVMS II		CVMS III		CVMS IV		CVMS V	
	M	F	M	F	M	F	M	F	M	F
	n=40	n=40	n=40	n=40	n=40	n=40	n=40	n=40	n=40	n=40
	Mean±S.D.	Mean±S.D.	Mean±S.D.	Mean±S.D.	Mean±S.D.	Mean±S.D.	Mean±S.D.	Mean±S.D.	Mean±S.D.	Mean±S.D.
	(median)	(median)	(median)	(median)	(median)	(median)	(median)	(median)	(median)	(median)
Age [Y]	10.08±3.14	9.85±3.01	11.06±3.13	10.70±3.04	12.16±1.82	11.72±3.06	16.06±3.10	15.22±2.89	17.29±3.48	16.96±3.37
	(9.33)	(9.25)	(9.33)	(9.17)	(12.13)	(10.75)	(16.46)	(15.71)	(18.50)	(18.54)
FMA [°]	29.79±4.84	29.47±5.52	26.45±5.06	29.01±5.54	28.03±5.20	27.00±4.56	26.85±4.94	29.11±4.55	30.22±6.92	31.01±7.09
	(30.05)	(29.30)	(30.00)	(28.00)	(27.70)	(27.05)	(27.35)	(28.80)	(29.70)	(32.35)
SNA [°]	79.99±3.22	81.77±3.19	80.61±3.22	81.07±3.39	80.97±3.86	81.64±3.19	81.11±3.39	79.41±3.64	81.48±3.34	80.93±2.97
	(80.25)	(82.25)	(80.15)	(82.25)	(81.66)	(82.15)	(81.05)	(79.35)	(81.30)	(81.05)
SNB [°]	76.73±3.44	76.95±3.90	76.78±3.58	77.37±4.16	76.59±4.31	78.59±3.65	79.67±4.60	76.31±4.61	80.19±4.74	78.49±4.87
	(76.85)	(76.65)	(76.90)	(77.10)	(76.10)	(78.22)	(79.05)	(75.30)	(80.05)	(78.55)

Sample size calculation

The sample size was calculated using G*Power software (latest version: 3.1.9.7; Heinrich-Heine-Universität Düsseldorf, Düsseldorf, Germany). A priori sample size estimation, performed at a 5% significance level (α = 0.05) and 80% power, revealed that a minimum of 40 patients per group was required. The experimental model described above was performed in the same way based on our previous research [10].

Outcome variables

**Figure 2:**
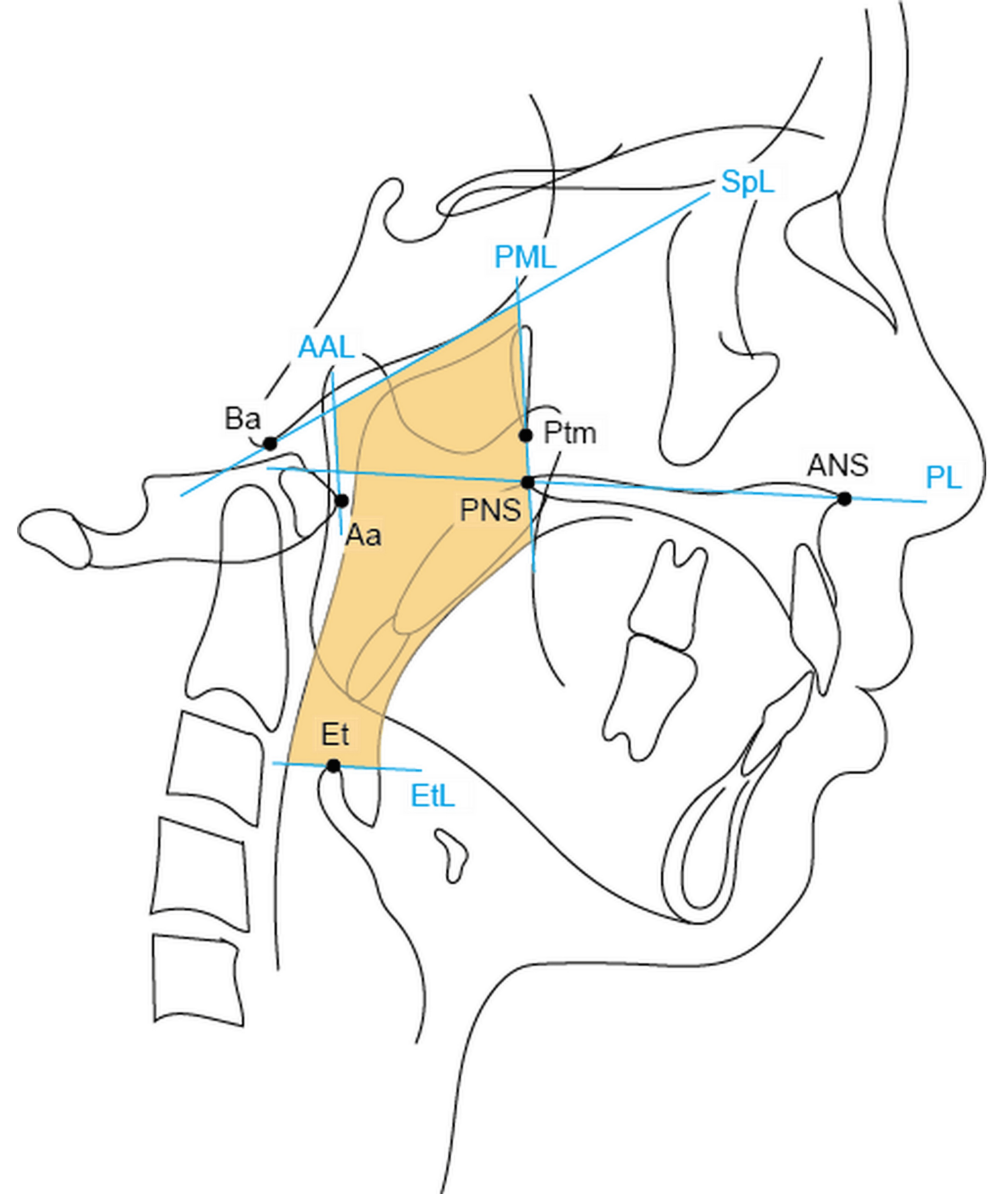
Cephalometric landmarks used to construct the two linear measurements and angular measurements analyzed in this study Linear parameters: ANS-PNS; distance between ANS and PNS, Ar-Go; distance between articulare and gonion, Go-Pog; distance between gonion and pogonion. Angular parameters: FMA; mandibular plane to Frankfort-Horizontal plane, SNA; sella-nasion to point A, SNB; sella-nasion to point B; ANS, anterior nasal spine; PNS, posterior nasal spine; PL, palatal line (line from the anterior nasal spine to the posterior nasal spine); SpL, sphenoid line (line tangential to the lower border of the sphenoid registered on the basion); PML, pterygomaxillary line (line perpendicular to the palatal line registered on the pterygomaxillon); Ptm, pterygomaxillary fissure; EtL, epiglottis line (line parallel to the palatal line registered on the most superior point on the epiglottis); AAL, anterior atlas line (line perpendicular to the palatal line registered on the anterior medial point of the atlas); Ba, basion; Aa, anterior medial point of the atlas; Et, epiglottis. This figure is the original work of the authors.

We traced the initial lateral cephalometric radiographs. The trace was plotted and measured using WinCeph ver.9 (Rise Corp., Tokyo, Japan) software. Measurement points are shown in Figure 2. Variables measured on each lateral cephalometric radiograph are shown in Table 2. The UA was defined as the trapezoidal region composed of the palatal line, sphenoid line, anterior atlas line, and pterygomaxillary line, plus the area outlined by the inferior border of the nasopharynx, the posterior surface of the soft palate, the posterior inferior surface of the tongue, the epiglottis line, and posterior pharyngeal wall, as shown in Figure 2. All lateral cephalometric radiographs were obtained according to international standards and the UA dimensions were measured using the same method as in the previous reports (Figure 2) [2,3,10].

**Table 2:** Definitions of measurement variables

Symbol	Description	Definition
S	Sella	The midpoint of the pituitary fossa
N	Nasion	The most anteroinferior point of frontal nasal suture
A	Point A	The deepest point on the curvature of the surface of the maxillary bone between ANS and the alveolar crest of the maxillary central incisor
B	Point B	The deepest point of the curved part of the mandibular alveolar process point
ANS	Anterior Nasal Spine	The cutting edge of the anterior nasal spine
PNS	Posterior Nasal Spine	The cutting edge of the posterior nasal spine
Ar	Articulare	Drafting intersection of mandibular process posterior margin and external skull base
Go	Gonion	Plotting intersection of the tangents of the mandibular ramus and body
Pog	Pogonion	The most prominent point of the mandibular chin ridge

Statistical analysis

The measurement was performed three times by one investigator (AM), and the average value was used as the measured value, but the evaluation was verified by another investigator (TI). Restoration extraction was performed using the bootstrap method with 1000 iterations to predict the population [3]. The measurement error in each measured value was calculated using Dahlberg's formula [13]. The Kruskal-Wallis test was performed for comparison between the groups, and the Steel-Dwass test was used for multiple comparisons (p<0.05). In addition, the weighted kappa coefficient was calculated based on the degree of intra-evaluator and inter-evaluator agreement to verify the reliability of the CVMS. The intra-evaluator agreement is based on two CVM stagings performed by one evaluator (AM) at three-week intervals. The degree of agreement between the evaluators is based on the CVM evaluation determined by each of the two evaluators (AM and TI). The main objective of this statistical analysis was to calculate standard values for the upper airway cross-sectional area, evaluate growth patterns, and assess the utility of the cervical vertebral maturation stage (CVMS) for growth assessments. To minimize research bias, random sampling was employed, and evaluations were verified by an independent reviewer, ensuring consistency and reliability of the findings. This approach helped minimize selection and measurement biases, enhancing the credibility of the study results. SPSS version 25 (Statistical Package of Social Sciences, IBM Corp., Armonk, NY, USA) was used for statistical analysis.

## Results

Area comparisons

**Figure 3:**
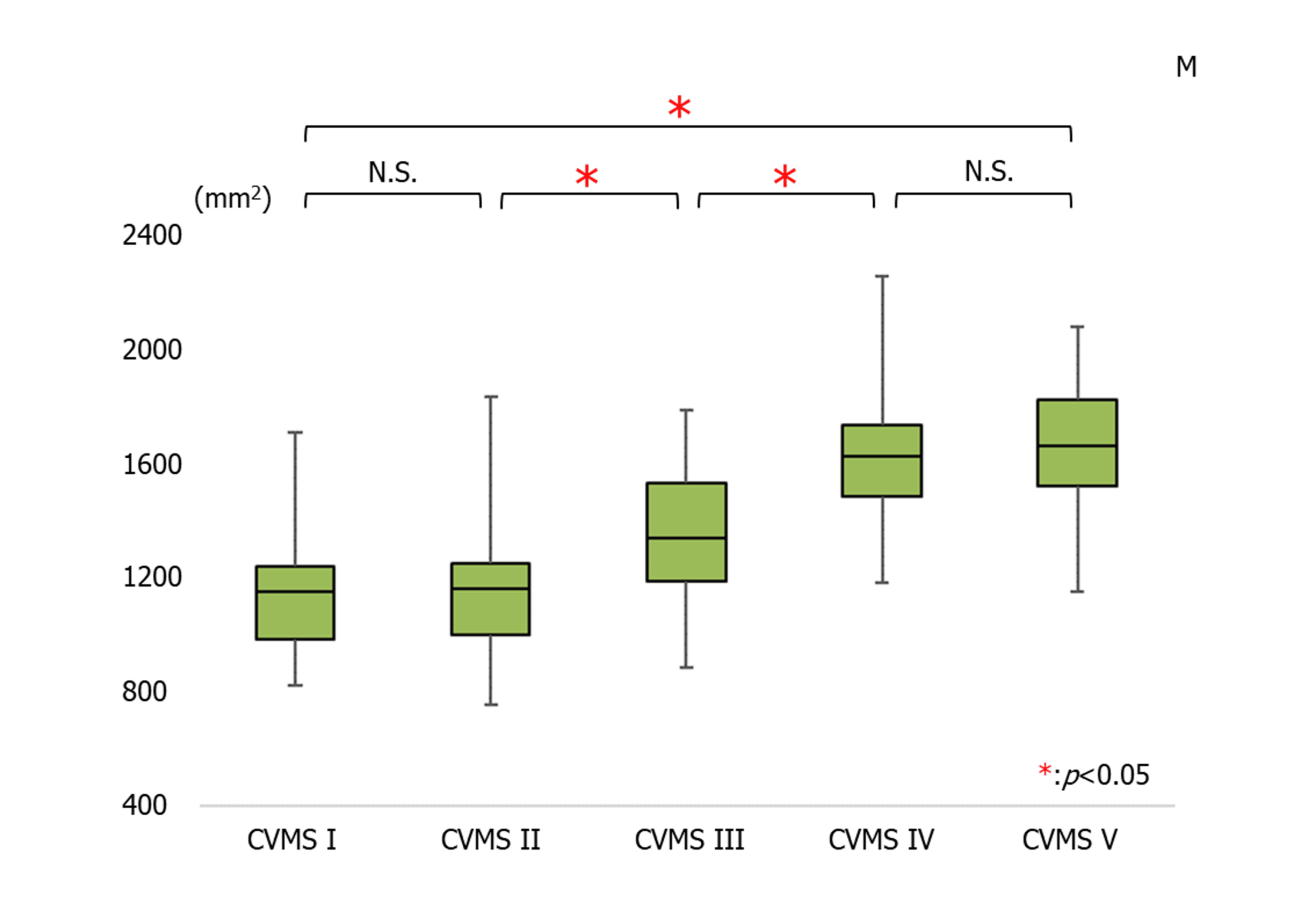
The average values for the airway in males (*p<0.05) CVMS, cervical vertebral maturation stage; N.S., not significant; M, male

The average cross-sectional area of the UA in males was 1144.16 ± 205.88 mm^2^ in the CVMS I group, 1148.82 ± 203.61 mm^2^ in the CVMS II group, 1357.84 ± 235.67 mm^2^ in the CVMS III group, 1642.78 ± 261.52 mm^2^ in the CVMS IV group, and 1662.05 ± 222.66 mm^2^ in the CVMS V group. There was a significant increase in the UA in CVMS V compared to CVMS I. There was a significant increase in CVMS III compared to CVMS II, and in CVMS IV compared to CVMS III (Figure 3).

**Figure 4:**
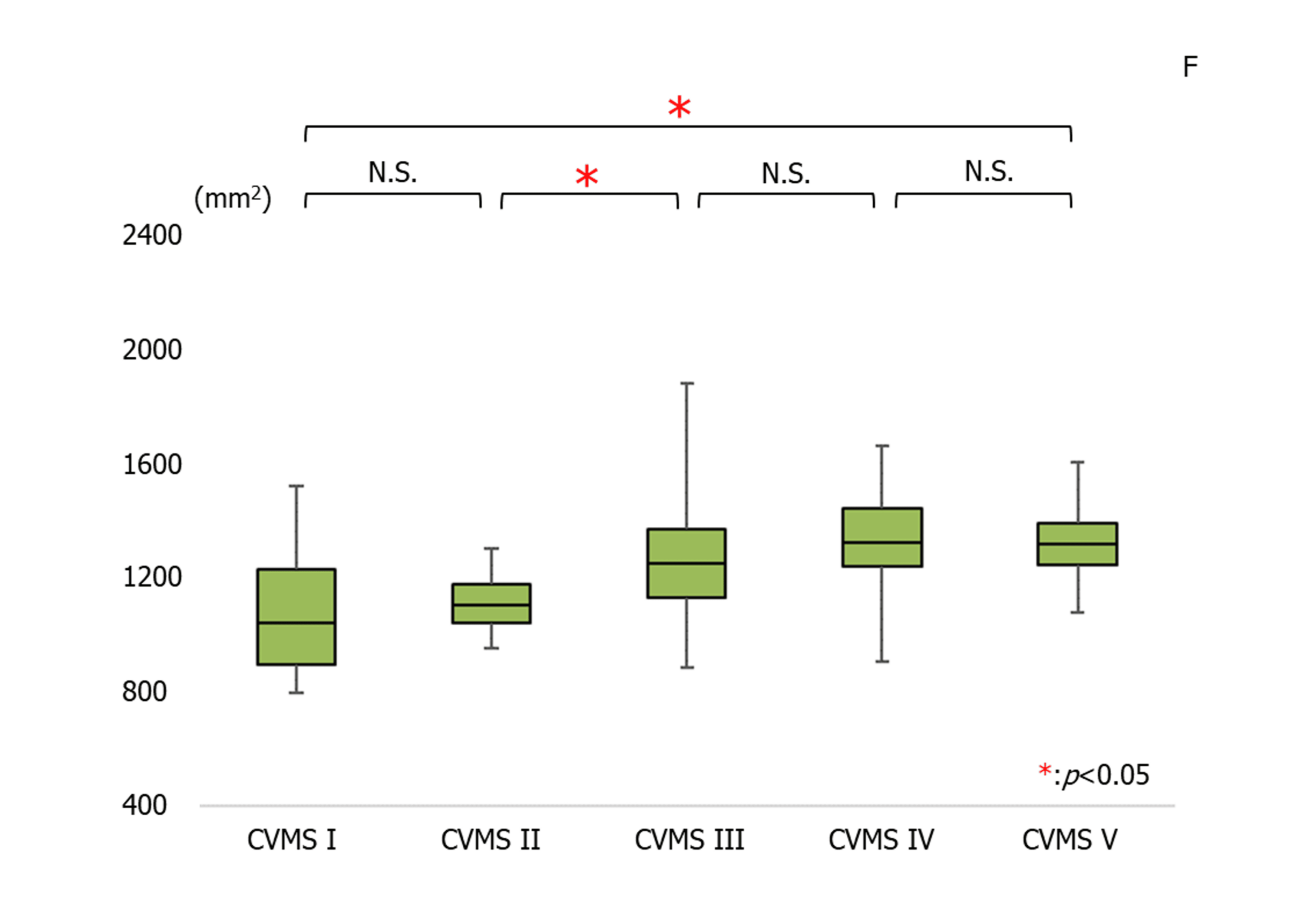
The average values for the airway in females (*p<0.05). CVMS, cervical vertebral maturation stage; N.S., not significant; F, female

On the other hand, the average cross-sectional area of the UA in females was 1071.29 ± 204.92 mm^2^ in the CVMS I group, 1112.43 ± 99.56 mm2 in the CVMS II group, 1260.83 ± 210.66 mm2 in the CVMS III group, 1344.69 ± 162.51 mm2 in the CVMS IV group, and 1327.93 ± 107.32 mm2 in the CVMS V group. There was a significant increase in the UA in CVMS V compared to CVMS I. There was a significant increase in the UA in CVMS III compared to CVMS II (Figure 4). There were significant differences in the timing of the UA growth pattern between males and females (Figures 3, 4).

The κ coefficient for the degree of intra-evaluator agreement was 0.88, and the κ coefficient for the degree of inter-evaluator agreement was 0.85, both showing almost perfect agreement.

The measurement error for the cross-sectional area of the UA was 3.14 mm^2^, as calculated using Dahlberg's formula, which was a sufficiently negligible error [13].

## Discussion

The previous cervical vertebral maturation (CVM) method was classified into six stages (CVs 1-6), but there were several issues. Notably, there was a requirement that only the cervical vertebrae visible even when the patient was wearing a protective radiation collar (C2, C3, C4) could be used for assessment. Furthermore, to facilitate clinical application and improve the accuracy of assessments, it was necessary to modify the method so that stages defined based on changes between stages in the conventional method could be determined from a single X-ray image [12]. To solve these issues, a new CVMS (CVMS I-V) was developed. In this study, we adopted the improved CVMS and conducted our research.

Now that we know the standard upper airway cross-sectional area values for each CVMS, we may be able to quantitatively point out the cause of obstructive apnea and snoring in children in terms of the size of the upper airway cross-sectional area as one cause and suggest a treatment for the above problems. In addition, physicians and dentists may be able to point out the cause of the problem in terms of the cross-sectional area of the upper airway. Doctors and dentists can also suggest to the above patients the exact timing of treatment to promote the growth of the upper airway based on the growth stages indexed by CVMS.

The findings of this study indicated that the size of the airway increases with growth in both males and females. For growing patients with large adenoids and tonsils who complain of snoring or OSA, medical doctors and dentists clarify that the size of the adenoids and tonsils decreases as they grow because the adenoids and tonsils are lymphoid tissue. However, our recent report showed that the adenoids and tonsils themselves do not rapidly regress with growth [3]. In other words, it should be explained that “the adenoids and tonsils appear large relative to the airway, but as the airway grows in size, the relative respiratory environment improves.”

When evaluating growth spurts of various organs of an individual, we use not only the patient’s chronological age but also the height, weight, and physiological age. Like carpal roentgens, cervical spine age is one of the indicators of physiological age [14-17]. We recently reported growth and development curves of the maxilla and mandible using cervical spine age [10]. The maxilla and mandible were reported to grow in both sexes. The airway also showed an almost similar trend of growth over time. This may indicate that the airway may enlarge in the same way that the maxilla and mandible grow: anteriorly and inferiorly.

The study showed that the cross-sectional area of the UA grows gradually and has a growth spurt between CVMS II and III and CVMS III and IV in males. It is known that the maxillary bone in males grows anteriorly and inferiorly, indexed by chronological age [18]. Our recent report suggested that anteroposterior growth of the maxilla between CVMS II and III reflects the timing of the maxillary growth spurt, which may be the reason for the enlargement of the airway between CVMS II and III in males [10]. The mandible, like the maxilla, grows anteriorly and downward, indexed by chronological age [18]. In our previous reports, males were observed to have a growth spurt in mandibular depth and length between CVMS III and IV [10]. We considered that anteroinferior movement of the lingual dorsum caused by the anterior migration of the lingual muscle is associated with the anteroinferior growth of the mandibular alveolar bone (the chin spine), which enlarges the UA [19,20]. It was suggested that the UA in males grows significantly when the maxilla and mandible grow.

It has been shown that the cross-sectional area of the UA grows gradually in females and that there is a growth spurt period between CVMS II and III. In our previous study, there was no clear growth spurt in anteroposterior maxillary length in females between CVMS II and III [10]. However, it is also known that the maxillary and mandibular bone in females gradually grows anteroinferiorly, indexed by chronological age [10,18]. The reason for the growth spurt between CVMS II and III in the female UA may be the particularly downward movement of the maxillary bone alongside the enlargement of the UA.

Based on the present results and our recent reports, we found that there is an association between UA growth and jaw growth, and that the timing of the growth spurt is more accurate when CVMS is used as an indicator [10]. Orthodontists often use functional appliances, such as the twin block (TB) or the maxillary protracting appliance (MPA), for patients with retrognathia and/or maxillary hypoplasia who are in their growth spurt to promote maxillo-mandibular growth [21]. We believe that our results further support the previous reports that the functional appliances used in orthodontic treatment, such as TB or MPA, promote not only maxilla-mandibular growth but also UA growth [18,22,23]. The results of the present study clarified the timing of the growth spurt of the maxilla, mandible, and UA and the optimal timing in the use of the functional appliance, which can help significantly increase the effectiveness of the treatment. Therefore, orthodontists should consider both occlusion and breathing environment in the treatment plan to ensure patient benefits.

One limitation of this study is that it is a cross-sectional survey, making it difficult to evaluate the continuous growth of the same individual. It would be desirable to conduct a longitudinal study as well and to judge the results of both cross-sectional and longitudinal studies from multiple perspectives. However, it may be unethical to take cephalometric images of patients not undergoing orthodontic treatment for the purpose of the above study. In addition, the growth of the airway of patients undergoing orthodontic treatment would not reflect the natural growth pattern of the airway. Therefore, it is difficult to conduct a continuous longitudinal study of individuals. However, cross-sectional surveys are widely used to obtain standardized values because there are fewer individual differences and a larger number of subjects compared to longitudinal surveys [2,24]. It is also widely accepted that growth patterns can be determined from these results. The method and results of this study are considered to be the best available at present. Second, we used two-dimensional lateral cephalometric radiography, but there is no concern about additional radiological exposure because lateral cephalometric radiography is routinely conducted in orthodontic diagnosis and treatment. Moreover, standardized lateral cephalometric radiographs are highly reproducible because the source-to-subject-to-film distance is kept strictly constant. The head position is also fixed. This allows the orthodontist to superimpose the cephalometric radiography within millimeters for the treatment/growth assessment of the patients [25-27]. Cone-beam computed tomography (CBCT) imaging still exposes the patient to more radiation than conventional cephalograms. Therefore, current guidelines do not support CBCT imaging as the routine modality for orthodontic practice. A previous study reported that the evaluation of the two-dimensional upper airway area by lateral cephalometric analysis correlates well with the three-dimensional upper airway assessment, and it can be used as a screening test to predict airway volume by computed tomography [28]. Therefore, we considered the methodology and results of this study to be meaningful and to contribute to the health of patients.

## Conclusions

The standard values of the UA cross-sectional area were calculated, and UA growth patterns in the Japanese population were clarified according to sex. There were differences in UA growth patterns between men and women. Moreover, the CVMS is a useful method for the UA growth assessment in Japanese patients. 
